# Facile Synthesis of Ultra-Small Silver Nanoparticles Stabilized on Carbon Nanospheres for the Etherification of Silanes

**DOI:** 10.3390/nano14131095

**Published:** 2024-06-26

**Authors:** Minghui Liu, He Huang, Changwei An, Xue Feng, Zijing Wang

**Affiliations:** 1 College of Biomedical and Chemical Engineering, Liaoning Institute of Science and Technology, No. 176 Xianghuai Road, Benxi 117004, China; anchangwei@lnist.edu.cn (C.A.); fengxue821@163.com (X.F.); zijingwang1990@163.com (Z.W.); 2 School of Petrochemical Engineering, Liaoning Shihua University, Fushun 113001, China

**Keywords:** Ag nanoparticles, stabilization, carbon nanospheres, silanes

## Abstract

The dehydrocoupling reaction between alcohols and hydrosilanes is considered to be one of the most atom-economical ways to produce Si–O coupling compounds because its byproduct is only hydrogen (H_2_), which make it extremely environmentally friendly. In past decades, various kinds of homogeneous catalysts for the dehydrocoupling of alcohols and hydrosilanes, such as transition metal complexes, alkaline earth metals, alkali metals, and noble metal complexes, have been reported for their good activity and selectivity. Nevertheless, the practical applications of these catalysts still remain unsatisfactory, which is mainly restricted by environmental impact and non-reusability. A facile and recyclable heterogeneous catalyst, ultra-small Ag nanoparticles supported on porous carbon (Ag/C) for the etherification of silanes, has been developed. It has high catalytic activity for the Si–O coupling reaction, and the apparent activation energy of the reaction is about 30 kJ/mol. The ultra-small Ag nanoparticles dispersed in the catalyst through the carrier C have an enrichment effect on all reactants, which makes the reactants reach the adsorption saturation state on the surface of Ag nanoparticles, thus accelerating the coupling reaction process and verifying that the kinetics of the reaction of the catalyst indicate a zero-grade reaction.

## 1. Introduction

Si–O coupling compounds are important chemical raw materials widely used as silanizing agents, surface modifiers, and inorganic–organic hybrid material [1,2,3,4]. They can also serve as a protecting group for hydroxyl functionality [5,6,7,8,9] in certain high-value organic chemicals. Among the various methods for producing Si–O coupling compounds, the dehydrogenative coupling between alcohols and hydrosilanes is the most commercially viable synthesis route. This is because the only byproduct of this reaction is hydrogen gas [10,11,12,13,14], making it economically and environmentally friendly and highly atom-economical.

Currently, reported catalysts for the dehydrogenative coupling of alcohols and hydrosilanes mainly include homogeneous catalysts such as transition metal complexes [3,15,16,17], alkaline earth metals, alkali metals [18,19,20], noble metal complexes (such as Rh, Ru, Ir, and Pd) [21,22,23], and heterogeneous d-Block Metal catalysts (such as Co, Ni, Cu, Zn, and Ag) [24,25]. Although these catalysts exhibit good catalytic activity and selectivity for the production of Si–O coupling compounds, they have drawbacks in terms of environmental pollution and high production costs, limiting their industrial applications from the perspectives of environmental protection and reusability. Therefore, the development of an environmentally friendly, cost-effective, and efficient heterogeneous catalyst for this reaction is highly desirable and urgent.

In recent years, metal nanoparticles have been widely studied [26,27] in the field of catalyst synthesis due to their unique properties such as small particle size, large surface area, and quantum confinement effect [28]. At the same time, carbon-based materials are considered to be excellent carriers due to their high stability and high porosity [29]. The introduction of heteroatoms (such as N and S) into carbon-based materials has been shown to modify the electronic structure of the local carrier, increase the functional reactivity of carbon sites [30], provide stable binding sites for metal active centers, and anchor metal nanoparticles, thereby enhancing the activity and prolonging the lifespan of the catalyst. As a result, carbon-based supported metal nanoparticle catalysts have made significant breakthroughs in various heterogeneous catalytic applications. A reported a novel N-doped carbon-based catalyst with silver and chromium nanostructures synthesized using chitosan as a precursor through biomass pyrolysis has been reported. [31] This catalyst has been used for the dehydrogenative coupling of various isomeric alcohols and hydrosilanes to produce silicon ethers. The catalyst exhibits excellent activity, good stability, and reusability. The mechanism of the silane alcohol dehydrogenation reaction was studied using in situ Raman spectroscopy.

A novel pre-polymer complexation strategy for the preparation of multiphase catalysts with porous carbon as the support and stably embedded ultra-small silver nanoparticles on the surface has been developed. By tuning the synthesis strategy, different sizes of Ag nanoparticles (with particle sizes of 2.3 ± 0.3 nm, 3.7 ± 0.5 nm, and 5.2 ± 0.8 nm) were obtained, while maintaining uniformity and high dispersion. Subsequently, the catalytic performance of this series of catalysts was evaluated for the dehydrogenative coupling of alcohols and silanes under mild conditions. As expected, the carbon-supported silver nanoparticles exhibited high activity and repeatability in the catalytic reaction. After ten cycles of use, the Ag nanoparticles on the carbon support did not aggregate or leak but always maintained high performance. Through catalyst structure characterization and reaction process evaluation, it was determined that the Ag nanoparticles in this type of catalyst act as active centers, like tiny “nanoreactors” [32], providing unique nano-scale reaction spaces that are isolated from the overall chemical environment, with the Ag nanoparticles restricted in the solid space at the nanoscale providing higher catalytic efficiency [33]. These findings may provide new ideas and more practical application prospects for catalytic reactions promoted by different metal nanoparticles.

## 2. Materials and Methods

### 2.1. Chemicals

All reagents were used as received without further purification. Melamine (C_3_H_6_N_6_), dimethylphenylsilane (C_8_H_12_Si, PhMe_2_SiH), 2,4-Diaminobenzenesulfonic acid (C_6_H_8_N_2_O_3_S), tetrahydrofuran (C_4_H_8_O), heptane (C_7_H_16_), methanol (MeOH), n-butanol (C_4_H_10_O), methyldiphenylsilane (C_13_H_14_Si, Ph_2_MeSiH), ethanol (EtOH), isopropanol (C_3_H_8_O), silver nitrate (AgNO_3_), triethylsilane (C_6_H_16_Si, Et_3_SiH), ammonium hydroxide (NH_3_·H_2_O, 25–28%), and formaldehyde solution (CH_2_O, 37–41%) were purchased from J&K Scientific (San Jose, CA, USA).

### 2.2. Preparation of Ag/C Catalysts

An amount of 0.252 g melamine (0.002 mol) and 0.75 g 40 wt% formaldehyde (0.01 mol) aqueous solution were added to a 100 mL round bottom bottle with vigorous stirring at 50 °C and 500 RPM until the melamine completely dissolved. Then, 0.07 mL 1 mol/L AgNO_3_ (0.00007 mol) and different amounts of 2,4-Diaminobenzenesulfonic acid (0.056 g/0.0003 mol, 0.094 g/0.0005 mol, and 0.131 g/0.0007) were added into the previous melamine prepolymer. After being mixed and stirred for 2 hours, the precipitate was recovered by centrifugation, washed with 20 wt% ethanol solution three times, and then dried in a vacuum oven at 100 °C for 10 h. Afterward, the powders were heated at 400 °C for 4 h in a fluent N_2_/H_2_ atmosphere (95%/5%). Finally, according to the amount of 2,4-Diaminobenzenesulfonic acid, we obtain the catalysts Ag/C-1, Ag/C-2, and Ag/C-3, or without adding AgNO_3_ and 2,4-Diaminobenzenesulfonic acid, the carrier carbon could be obtained [34].

### 2.3. Si–O Coupling Reaction

An amount of 6 mL tetrahydrofuran, 0.2 mL EtOH, and 0.1 mL (0.65 mmol) dimethylphenylsilane were added to a 20 mL reactor and fully ultrasound stirred until the mix was uniform. Then, the Ag/C catalyst (silver addition is 0.1 mol% silane) was added into the mixture and the product was detected by gas chromatography at room temperature. The internal standard was n-heptane. After the reaction, the catalyst was recovered by filtration. Then, it was washed three times with 5 mL of ethanol and dried overnight at 100 °C under an air atmosphere.

### 2.4. Kinetic Resolution through Si–O Coupling

An amount of 6 mL tetrahydrofuran, 1.0 mL EtOH, and 0.1 mL (0.65 mmol) dimethylphenylsilane were added to a 20 mL microreactor or 0.05 mL of EtOH and 1.0 mL (6.5 mmol) of dimethylphenylsilane was added to 6 mL of tetrahydrofuran, and the ultrasound was sufficient until the mix was homogeneous. Afterwards, the Ag/C catalyst (2.3 ± 0.3 nm) (the amount of silver added was 0.1 mol% of the reaction substrate) was added and monitored by gas chromatography for the reaction feedstocks (dimethylphenylsilane and ethanol) and the product (dimethylphenylethoxysilane) at a certain time interval. The internal standard was n-heptane.

### 2.5. Characterization

Transmission electron microscopy (TEM) images and energy dispersive X-ray spectroscopy (EDS) mapping were observed by a JEM-2000 instrument (JEOL, Tokyo, Japan) using an accelerating voltage of 120 kV. X-ray diffraction (XRD) patterns of Ag/C were determined via Rigaku DMAX IIIVC that had Cu-Kα (0.1542 nm) radiation with scanning at a rate of 8° min^−1^ in the 10° to 80° (2 theta). X-ray photoelectron spectroscopy (XPS) was performed by a Thermo Fisher Scientific Escalab 250Xi (Tokyo, Japan) with a monochromatic Mg Kα source. The reference of all the binding energies was the C1s peak at 284.6 eV. The detection of the etherification process was performed by Agilent 7000B triple quadrupole gas chromatography–mass spectrometry (GC-MS) (Agilent, Santa Clara, CA, USA). The silver load of all materials was determined by an inductively coupled plasma atomic emission spectrometer (ICP-AES, ICPE-9000, Shimadzu, Kyoto, Japan). An elemental analyzer (vario MACRO cube, Langenselbold, Germany) was used to quantitatively examine C, H, O, N, and S in Ag/C samples.

## 3. Results and Discussion

**Scheme 1:**
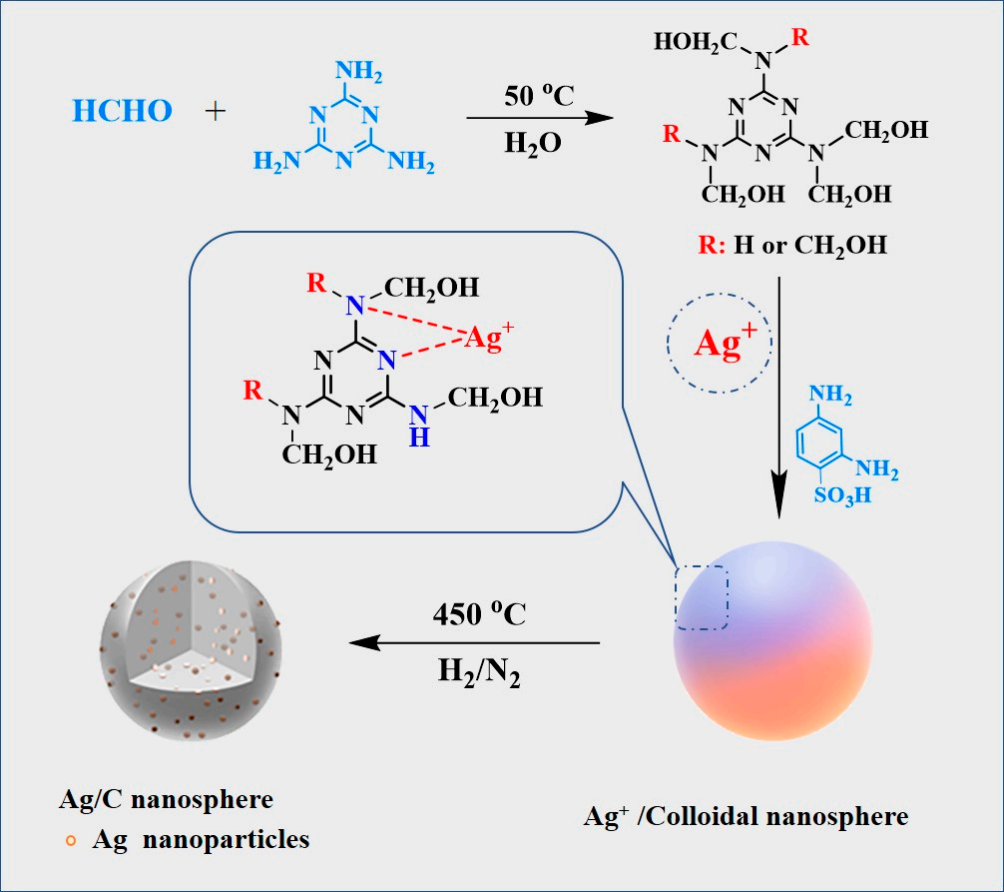
Schematic representation of the synthesis procedures of Ag/C catalysts.

The preparation method of the Ag/C catalyst is displayed in Scheme 1. In detail, a solution of melamine and formaldehyde were mixed to obtain melamine pre-polymer. Subsequently, a fixed mass of AgNO_3_ aqueous solution was added to the melamine prepolymer, followed by different masses of 2,4-diaminobenzenesulfonic acid. Characterization by electrospray ionization mass spectrometry (Figure S1) confirmed that Ag^+^ underwent complexation with the N atoms in the pre-polymer, forming Ag^+^ complexes which were dispersed in the reaction solution. Simultaneously, cross-linking between melamine pre-polymer molecules was achieved through the two amine groups in 2,4-diaminobenzenesulfonic acid. This cross-linking process, characterized by FT-IR (Figure S2) and solid-state ^13^C NMR (Figure S3), demonstrated that under the catalytic action of sulfonic acid groups, the –NHCH_2_OH in the pre-polymer structure dehydroxylated to form –NHCH_2_^+^ and underwent electrophilic substitution reactions with –NH_2_ or –NHCH_2_OH, resulting in the bridging of –NHCH_2_NH– and –NHCH_2_OCH_2_NH– linkages. Finally, the complexed Ag^+^ was uniformly encapsulated within the pre-polymer and condensed into colloidal nanospheres (Ag/MF). The subsequent high-temperature H_2_ reduction process yielded the catalytic material Ag/C with highly dispersed carbon spheres loaded with ultra-small Ag^0^ nanoparticles.

**Figure 1:**
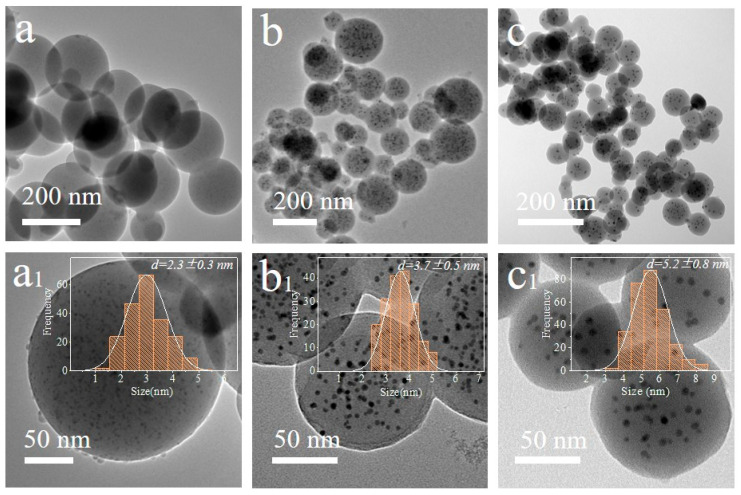
TEM images and corresponding size distribution of Ag/C-1 (**a**,**a_1_**), Ag/C-2 (**b**,**b_1_**), and Ag/C-3 (**c**,**c_1_**).

The TEM images and corresponding size distribution of Ag/C-1 (Figure 1a,a_1_), Ag/C-2 (Figure 1b,b_1_), and Ag/C-3 (Figure 1c,c_1_) are observed, and it is found that with the increasing amount of 2,4-diaminobenzenesulfonic acid, the colloidal microspheres maintain their uniform dispersion as spherical particles after calcination and reduction, but their sizes gradually decrease [34] (Figure S4). Nevertheless, all the spherical microspheres are uniformly loaded with highly dispersed Ag nanoparticles, with particle sizes of 2.3 ± 0.3 nm, 3.7 ± 0.5 nm, and 5.2 ± 0.8 nm. Additionally, elemental analysis by inductively coupled plasma atomic emission spectroscopy (ICP-AES) reveals that the Ag loading amounts in the catalysts are 3.63 wt%, 3.35 wt%, and 3.28 wt%, respectively. This indicates that the addition of 2,4-diaminobenzenesulfonic acid not only influences the sizes of colloidal microspheres and Ag nanoparticles but also slightly affects the Ag loading amount, which in turn have varying degrees of impact on the catalytic activity.

**Figure 2:**
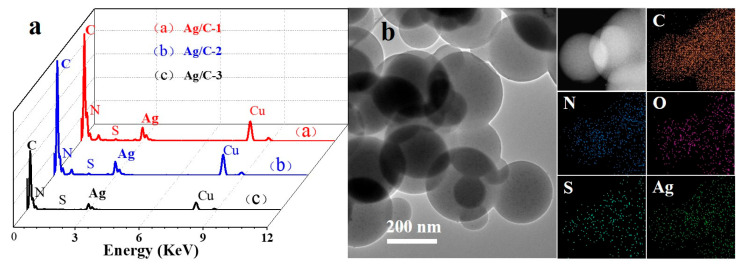
EDS spectra (**a**) of all Ag/C catalysts; the element mapping images (**b**) for C, O, N, S, and Ag of Ag/C-1.

The Energy Dispersive Spectrometer (EDS) (Figure 2a) and combustion elemental (Table S1) analysis of catalyst reveal the presence of elements C, N, S, Cu, and Ag in all three catalyst spectra. Specifically, C, N, and S originate from melamine formaldehyde prepolymers and 2,4-diaminobenzenesulfonic acid, Cu is derived from the copper mesh, and Ag serves as the catalytic active center. Meanwhile, X-ray energy spectrum element mapping (EDS Mapping) analysis was performed on Ag/C-1 (Figure 2b), demonstrating that C, N, and S are distributed within the colloidal microspheres, while Ag existed as dispersed nanoparticles. Additionally, the adsorption–desorption isotherms of Ag/C samples exhibit distinct hysteresis loops, which fall under the category of typical type II isotherms (Figure S5), indicating that the material is indeed related to either non-porous or macro-porous materials. The pore size distribution data showed average pore sizes in the range of 6.2–18.8 nm (Table S2), which is due to the stacking of pores between the spheres of Ag/C samples. Compared to Ag/C-2 (126.7 m^2^·g^−1^) and Ag/C-3 (100.7 m^2^·g^−1^), Ag/C-1 boasts a higher specific surface area of 130.8 m^2^·g^−1^ (Table S2). Encouragingly, there is a large specific surface area of Ag/C-1 with more active sites and more ion transport channels, which is conducive to contact between the catalyst and substrate, which could enhance the catalytic activity. The results indicated that the adsorption–desorption isotherms of Ag/C-1, Ag/C-2, and Ag/C-3 were all type II isotherms, representing the typical physical adsorption process on non-porous adsorbents. This finding suggests that Ag nanoparticles are evenly dispersed on the surface of colloidal microspheres.

**Figure 3:**
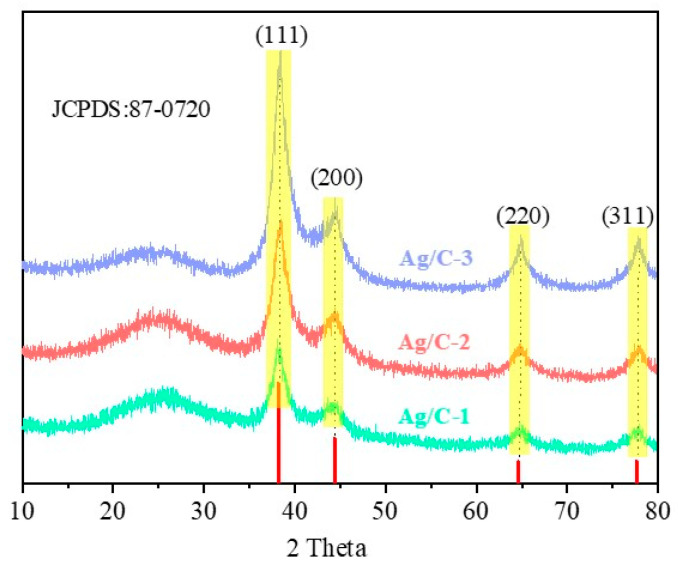
XRD patterns of all Ag/C catalysts.

Figure 3 displays the XRD patterns of the as-prepared Ag/C catalysts. The four distinct characteristic peaks appear at 2θ = 38°, 44°, 65°, and 82° and can be assigned to the (111), (200), (220), and (311) crystal planes of elemental silver (JCPDS No.87-0720), respectively, which proves that Ag nanoparticles have been reduced to zero valence. Moreover, as the size of Ag nanoparticles increases, the intensity of the four characteristic peaks also gradually increases. The Ag nanoparticle size calculated by Scherer’s formula is consistent with the Ag nanoparticle size calculated by TEM image statistics. A broad peak between 17° and 28° was regarded as the (002) plane of the amorphous carbon; no other peaks could be observed, indicating that the reduction process did not change the crystalline phase of Ag and C.

**Figure 4:**
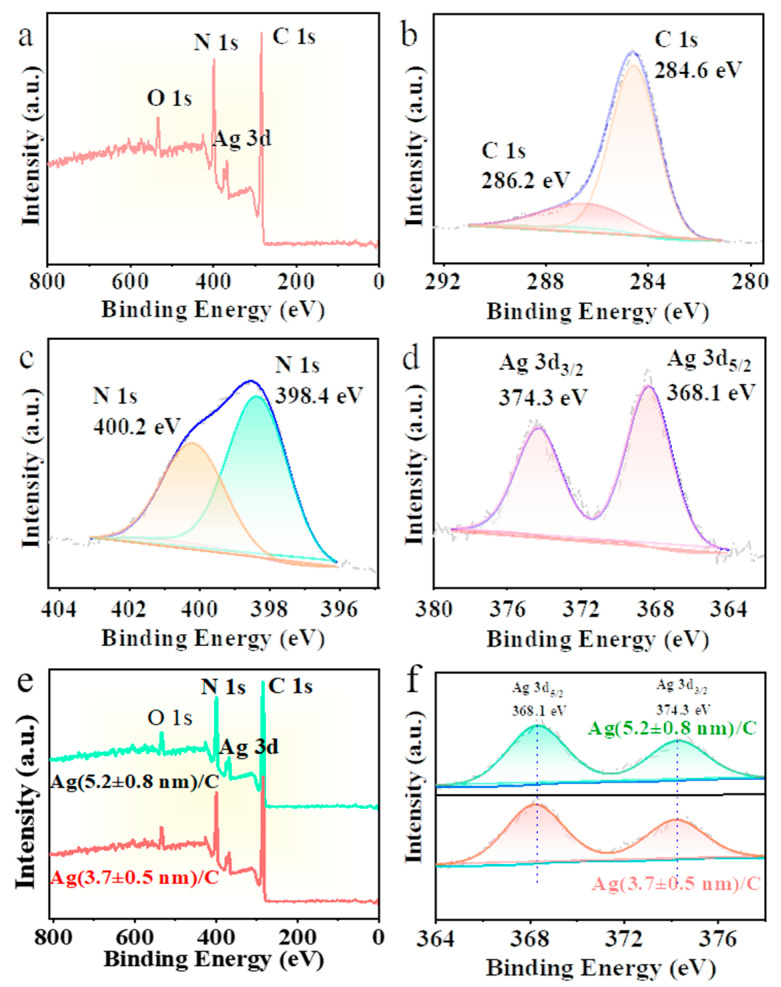
XPS spectra of (**a**) Ag/C-1, (**b**) C 1s, (**c**) N 1s, and (**d**) Ag 3d; (**e**) XPS survey spectrum and (**f**) Ag 3d of Ag/C-2 and Ag/C-3.

Ag/C catalysts were characterized by XPS, and the results are shown in Figure 4. Figure 4a presents the XPS wide-scan spectrum of Ag/C-1, in which the C, N, Ag, and O elements are present in the Ag/C catalysts. Due to the low sulfur content, the S element was not detected. Figure 4b shows the C 1s binding energy of Ag/C-1, with peaks at 284.6 eV and 286.2 eV representing the binding energies of C–C and C–N, respectively. In Figure 4c, the two peaks of N 1s correspond to pyridinic N (398.4 eV) and pyrrolic N (400.2 eV). The two peaks at 368.1 eV and 373.3 eV in Figure 4d represent Ag 3d_5/2_ and Ag 3d_3/2_, respectively, indicating that the Ag element in this binding energy is in the zero-valent state. Subsequently, XPS wide-scan (Figure 4e) and narrow-scan analysis of Ag 3d (Figure 4f) were also conducted on the Ag/C-2 and Ag/C-3 catalysts, showing consistent element distribution and valence states with Ag/C-1. This suggests that the three catalysts have the same elemental composition and electronic configuration of catalytically active centers.

Prior to the catalytic performance of the Si–O coupling reaction of the prepared Ag/C catalysts, blank experiments involving only ethanol and dimethylphenylsilane were conducted without the use of any catalyst (Table 1, Entry 1), revealing no occurrence of the Si–O coupling reaction. Subsequently, the carrier carbon was used as the control catalyst group, and three catalysts, Ag/C-1, Ag/C-2, and Ag/C-3, were compared for the Si–O coupling reaction of ethanol and dimethylphenylsilane. The results showed that the activated carbon in the control group (Table 1, Entry 2) exhibited a minimal Si–O coupling reaction under the same reaction conditions, with a dimethylphenyl ethoxy silane yield of only 1.4%, demonstrating the lack of activity of a single C-based carrier for catalyzing the Si–O coupling reaction. However, the catalysts Ag/C-1, Ag/C-2, and Ag/C-3 all exhibited high catalytic activity for the Si–O coupling reaction, with higher selectivity and conversion of dimethylphenylsilane within a certain period of time (Table 1, Entrys 4–6). Particularly, Ag/C-1 (Table 1, Entry 4) achieved a selectivity and conversion rate of over 99% and 98.9% for dimethylphenylsilane into ethoxydimethylphenylsilane after 120 min of reaction, attributed to the large specific surface area, good pore size distribution and small particle size. A large specific surface area and good pore size distribution are conducive to contact between the catalyst and substrate, which improves the catalytic activity. Importantly, GC-MS analysis (Figure S6) revealed ethoxydimethylphenylsilane as the only observed compound without any other byproducts. The calculated turnover frequency (TOF) values indicated the highest TOF value for Ag/C-1 at 6.37 min^−1^, followed by Ag/C-2 at 4.97 min^−1^ and Ag/C-3 at 3.18 min^−1^. 

**Figure 5:**
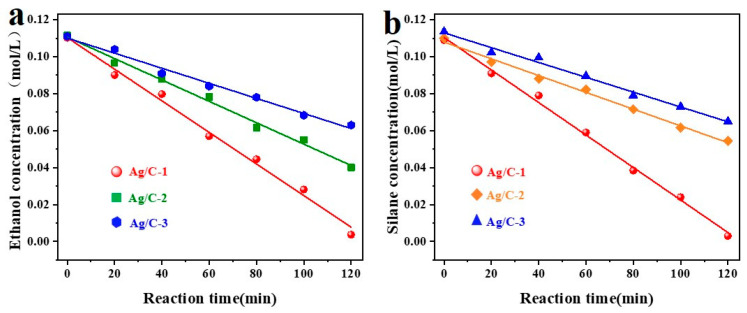
Kinetic analysis of Si–O coupling catalyzed by different catalysts on ethanol (**a**) and silane (**b**), reaction conditions: 6 mL tetrahydrofuran, 1.0 mL EtOH, 0.1 mL dimethylphenylsilane, and Ag/C catalyst (2.3 ± 0.3 nm) (the amount of silver added was 0.1 mol% of the reaction substrate) were added to a 20 mL microreactor under ultrasonicaing (120 min), and monitored by gas chromatography at a certain time interval.

The kinetics of the coupling reaction between dimethylphenylsilane and ethanol catalyzed by Ag/C were investigated. Standard working curves were constructed for both reactants, as shown in Figure S6. Subsequently, the kinetics of the coupling reaction using three different Ag/C catalysts were analyzed, with the results presented in Figure 5. Figure 5a illustrates the kinetic curves of ethanol concentration under the influence of the three catalysts, showing a linear relationship between ethanol concentration and reaction time, indicating zero-order kinetics for the Si–O coupling reaction. In Figure 5b, a linear relationship between dimethylphenylsilane concentration and time was observed, suggesting zero-order kinetics for dimethylphenylsilane. The kinetic constants and the determination coefficients are shown in Table S3. This suggests that the total reaction order of the Ag/C catalysts for the coupling reaction of dimethylphenylsilane and ethanol is zero-order.

The two substrates can always be in a high concentration state on the surface of the catalyst’s active center, because the reaction rate of the zero-order reaction is always constant. The catalytic reaction can still ensure enough effective collisions to maintain a fixed catalytic rate when the substrate concentration is reduced, as the porous structure of carrier C has a strong enrichment effect on the reactants. This conjecture was confirmed through adsorption studies; pertinent adsorption characteristics data are displayed in Tables S4 and S5. The adsorption capacity of the catalyst for silane is about 1.5 mmol/g, and the adsorption capacity for ethanol is about 1.1 mmol/g, which confirms that the catalyst can indeed enrich the two substrates. 

**Figure 6:**
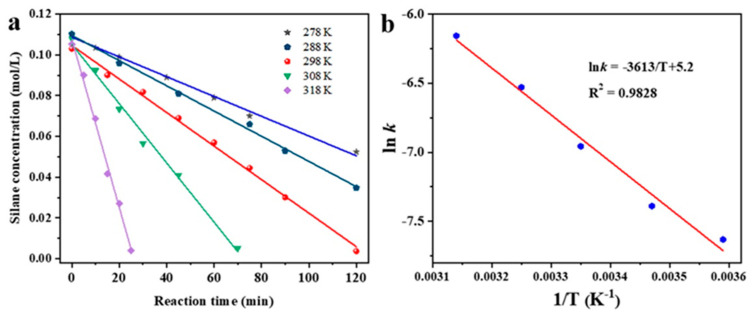
Kinetics of the coupling of silane at 278 K, 288 K, 298 K, 308 K, and 318 K (**a**) and Arrhenius plot for the coupling reaction catalyzed by Ag/C-1 (**b**).

Based on the reaction kinetics analysis of the three catalysts, it was found that the Ag/C-1 catalyst exhibited the highest catalytic activity. Subsequently, a more detailed calculation of its activation energy was conducted to elucidate its superior catalytic performance. The calculation of activation energy required an initial determination of reaction rate constants under different reaction temperature conditions. Figure 6a shows the kinetic curves of the coupling reaction between dimethylphenylsilane and excess ethanol catalyzed by Ag/C-1 at 278 K, 288 K, 298 K, 308 K, and 318 K. It can be observed that within the temperature range of 278 K to 318 K, the concentration of dimethylphenylsilane exhibited a good linear relationship with reaction time, indicating zero-order kinetics of the Si–O coupling reaction under excess ethanol conditions. The slope of each line corresponds to the reaction rate constant at the respective temperature conditions.

The relationship between activation energy, reaction rate constant, and temperature follows the Arrhenius equation:

(1) $$\mathrm{Ln}k=\mathrm{Ln}A-\frac{E_{a}}{\mathrm{RT}}$$

where *k* is the reaction rate constant at temperature *T*, *A* is the pre-exponential factor independent of temperature T, *E_a_* represents the apparent activation energy, and *R* is the molar gas constant 8.314 J/(mol·K).

The relationship between the reaction rate constant *k* and the corresponding temperature *T* obtained from the Arrhenius equation is expressed as follows:

(2) $$\mathrm{Ln}k=-\frac{3613}{T}+5.2$$

The linear correlation coefficient is 0.9828 (Figure 6b).

Therefore, the apparent activation energy *E_a_* for the coupling reaction of dimethylphenylsilane and ethanol catalyzed by Ag/C-1 is about 30 kJ/mol, indicating that it has a lower activation energy and a higher catalytic activity [22,35,36].

**Figure 7:**
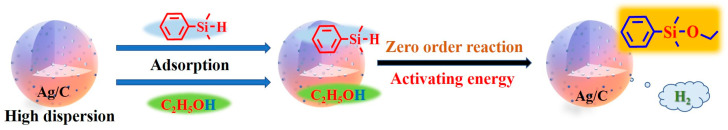
Proposed pathway for the Si–O coupling reaction on Ag/C-1.

Through the characterization of catalysts and kinetic studies, we speculate on the possible reaction pathway of the coupling reaction of dimethylphenylsilane and ethanol catalyzed by Ag/C, as illustrated in Figure 7: Initially, the porous structure of the catalyst has an enriching effect on both reactants. Ag nanoparticles’ surfaces can become saturated with both ethanol and dimethylphenylsilane. Ag nanoparticles’ surfaces activated the two reactants, causing the coupling process to occur [36]. Due to the catalytic reaction being confirmed as a zero-order reaction kinetic mechanism, the catalytic process of Ag nanoparticles represents the rate-controlling step of the overall reaction [37]. The catalyst Ag/C-1 with the smallest particle size has a higher proportion of surface atoms, meaning a higher number of low-coordination atoms, enabling the activation of more dimethylphenylsilane and ethanol molecules. Thus the overall reaction process is accelerated and the catalytic activity is the highest.

**Figure 8:**
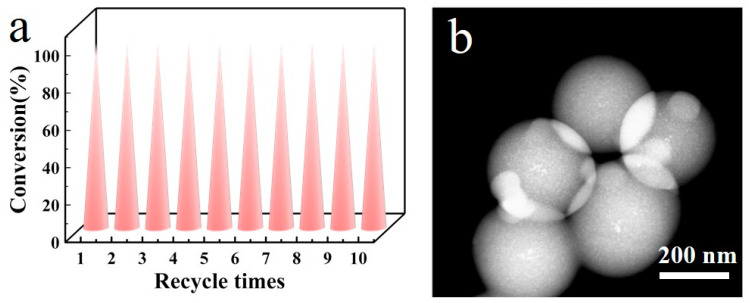
Recycling tests for 10 cycles of Ag/C-1 (**a**) and STEM image after 10 cycles of Ag/C-1 (**b**). Reaction conditions: 6 mL tetrahydrofuran, 0.2 mL EtOH, 0.1 mL dimethylphenylsilane, and Ag/C catalyst (2.3 ± 0.3 nm) (the amount of silver added was 0.1 mol% of the reaction substrate) were added to a 20 mL microreactor, ultrasonically mixed for 120 min, and monitored by gas chromatography at a certain time interval.

Due to the catalyst being a heterogeneous catalyst, its solid powder can be easily separated and recovered from the liquid-phase reaction system through centrifugal filtration after a single reaction. Therefore, we conducted 10 cycles of repeated use of Ag/C-1, and the reaction results are shown in Figure 8. After 10 reactions, the conversion rate of the catalyst remained above 98%, indicating the excellent catalytic activity of the catalyst. Characterization by STEM revealed that after 10 reactions, the Ag nanoparticles in the catalyst Ag/C-1 did not exhibit significant aggregation and loss, indicating excellent recyclability of the catalyst. These test results play a key role in the practical application promotion of the catalyst.

The above research demonstrated that the catalyst Ag/C-1 exhibits outstanding catalytic performance in the coupling reaction of dimethylphenylsilane and ethanol. Subsequently, an attempt was made to expand the variety of reactants by replacing dimethylphenylsilane with various silanes and ethanol with different alcohols. The results of these reactions are presented in Table 2. Analysis of the data in the table reveals that coupling reactions with small-molecule alcohols (such as methanol) and various silanes exhibit faster reaction rates (Table 2 entries 1–3), particularly with dimethylphenylsilane (Table 2 entry 1) where complete conversion is achieved in just 1 hour with a high TOF value of 9.37 min^−1^. When using n-butanol, the coupling reaction is conducted at 50 °C (Table 2 entries 7 and 8) with lower reaction rates, while coupling with larger molecules such as benzyl alcohol requires conditions at 80 °C (Table 2 entries 9 and 10) resulting in decreased reaction activity. Particularly for the reaction between bulky diphenylmethylsilane and benzyl alcohol, limited by steric hindrance [38], the conversion rate only reaches 5%. Nevertheless, the catalyst Ag/C-1 has demonstrated its ability to catalyze the Si–O coupling reaction of various silanes and alcohols, leading to the generation of silane ether, thus providing new research perspectives and broad application prospects in Si–O coupling reactions for silane ether production.

## 4. Conclusions

In summary, we report a carbon-based supported ultra-small silver nanoparticle heterogeneous catalyst (Ag/C) for the siloxane-alcohol coupling reaction. The synthesized catalyst was characterized by various techniques, confirming its configuration of highly dispersed ultra-small silver nanoparticles on porous carbon spheres. Furthermore, kinetic analysis of the coupling reaction between dimethylphenylsilane and ethanol demonstrated that the Ag/C catalyst exhibited zero-order kinetics for such reactions, due to the fact that the porous structure of the carbon can have a strong enrichment effect on the two substrates, resulting in the saturation state on the surface of the active center Ag nanoparticles. According to the Arrhenius formula, the apparent activation energy of the Ag/C-1 reaction is about 30 kJ/mol, it has excellent catalytic activity, and the activity was almost constant after 10 cycles. Our results suggest that this carbon-based supported Ag catalyst provides a new research direction and broad application prospects for the siloxane-alcohol coupling reaction in the synthesis of silane-based ethers.

## Figures and Tables

**Table 1:**
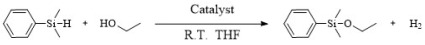
Si–O coupling reaction with different catalysts.

Entry ^a^	Catalyst	Time (min)	Selectivity ^b^ (%)	Conversion ^b^ (%)	TOF ^c^
1	None	160	0	0	-
2	C	160	0	1.4	-
3	AgNO_3_	160	98.2	13.7	-
4	Ag/C-1	120	99.3	99.1	6.37
5	Ag/C-2	135	99.2	98.2	4.97
6	Ag/C-3	160	99	98.9	3.18

^a^ Typical reaction conditions: 6 mL tetrahydrofuran (THF) as solvent, 0.1 mL dimethylphenylsilane, 0.2 mL ethanol, catalyst (0.1 mol% Ag), stirring rate of 500 rpm at R.T. ^b^ Selectivity (%) and Conversion (Con.%) were determined and calculated by gas chromatography at room temperature, using formulae M_products_/(M_0_-M_1_) and (M_0_-M_1_)/M_0_, where M_0_ and M_1_ are the amounts of dimethylphenylsilane before and after a typical reaction. ^c^ TOF = TON/T, TON = N_s_/N_c_, N_s_ is the amount of dimethylphenylsilane in the reaction calculated via the formula Con.%*N_0_, N_0_ is the amount of dimethylphenylsilane before reaction, N_c_ is the amount of Ag, calculated via the formula m_c_*wt%/108, m_c_ is the mass of Ag/C, wt% is the mass fraction of Ag, and T is the catalyst life time.

**Table 2:** Si–O coupling of different silanes and alcohols catalyzed by Ag/C-1.

Entry ^a^	Silane	Alcohol	Temperature (°C)	Time (h)	Selectivity ^b^ (%)	Conversion ^b^ (%)	TOF ^c^
1	PhMe_2_SiH	Methanol	25	1	99.1	99.2	9.37
2	Ph_2_MeSiH	Methanol	25	1.1	98.8	98.5	9.10
3	Et_3_SiH	Methanol	25	1.5	99.2	99.3	7.33
4	PhMe_2_SiH	Ethanol	25	2	98.9	98.7	6.37
5	Ph_2_MeSiH	Ethanol	25	2.5	99.1	98.8	2.43
6	Et_3_SiH	Ethanol	25	7	98.5	96.5	1.05
7	PhMe_2_SiH	*n*-Butanol	50	5	99.5	99.5	1.57
8	Ph_2_MeSiH	*n*-Butanol	50	6	99.3	98.3	1.22
9	Et_3_SiH	Benzyl alcohol	80	10	83.7	91.7	0.77
10	Ph_2_MeSiH	Benzyl alcohol	80	10	2	5.1	0

^a^ Typical reaction conditions: 6 mL tetrahydrofuran (THF) as solvent, 0.1 mL alkylsilane, 0.2 mL alcohol, catalyst (0.1 mol% Ag), stirring rate of 500 rpm at R.T. ^b^ Selectivity (%) and Conversion (Con.%) were determined and calculated by gas chromatography at room temperature, with the formulae M_products_/(M_0_-M_1_) and (M_0_-M_1_)/M_0_, where M_0_ and M_1_ are the amounts of dimethylphenylsilane before and after a typical reaction. ^c^ TOF = TON/T, TON = N_s_/N_c_, N_s_ is the amount of alkylsilane during the reaction calculated by Con.%*N_0_, Con.% is the conversion of alkylsilane, N_0_ is the amount of alkylsilane before the reaction, N_c_ is the amount of Ag calculated by m_c_*wt%/108, where m_c_ is the mass of the catalyst, wt% is the mass fraction of Ag determined by ICP, and 108 is the molar mass of Ag. T is the catalyst life time.

## Data Availability

Data are contained within the article and Supplementary Materials.

## Supplementary Materials

The following are available online at https://www.mdpi.com/article/10.3390/nano14131095/s1, Figure S1: ESI mass spectrum of Ag^+^ complex; Figure S2: FT-IR spectra of Ag/MF colloidal nanospheres; Figure S3: ^13^C NMR spectra of Ag/MF colloidal nanospheres; Figure S4: SEM images of Ag/MF colloidal nanospheres at different concentrations of 2,4-diaminobenzenesulfonic acid (a: Ag/MF-1, b: Ag/MF-2, c: Ag/MF-3); Figure S5: N_2_ adsorption/desorption isotherms of Ag/C; Figure S6: GS-MS of Si–O coupling reaction; Figure S7: Kinetic plots of ethanol (a) and silane (b) in Si–O coupling reaction; Table S1: Combustion elemental analysis of Ag/C; Table S2: Specific surface area and porosity of Ag/C; Table S3: Summary kinetic parameters for zero-grade Kinetic model; Table S4: Adsorption amount of ethanol over Ag/C; Table S5: Adsorption amount of silane over Ag/C.
