# Supplementary material for: Facile Synthesis of Ultra-Small Silver Nanoparticles Stabilized on Carbon Nanospheres for the Etherification of Silanes
Source: Nanomaterials (Basel). 2024 Jun 26;14(13):1095. doi: 10.3390/nano14131095 (PMC11243459; doi:10.3390/nano14131095)
Supplement: Supplementary file 1 [file nanomaterials-14-01095-s001.zip › nanomaterials-3028320-supplementary.pdf]

nanomaterials

Supporting information

Facile Synthesis of Ultra Small Silver Nanoparticles Stabilized on Carbon Nanospheres for the Etherification of Silanes

Minghui Liu[1,*], He Huang[2,*], Changwei An[1], Xue Feng[1], Zijing Wang[1]

1 College of Biomedical and Chemical Engineering, Liaoning Institute of Science and Technology, No. 176 Xianghuai Road, Benxi 117004, Liaoning, P. R. China
2 School of Petrochemical Engineering, Liaoning Shihua University, Fushun 113001, Liaoning, P. R. China

* Correspondence: Dadou_1117@163.com; huanghe@lnpu.edu.cn

Supporting Figures (S1 to S6)

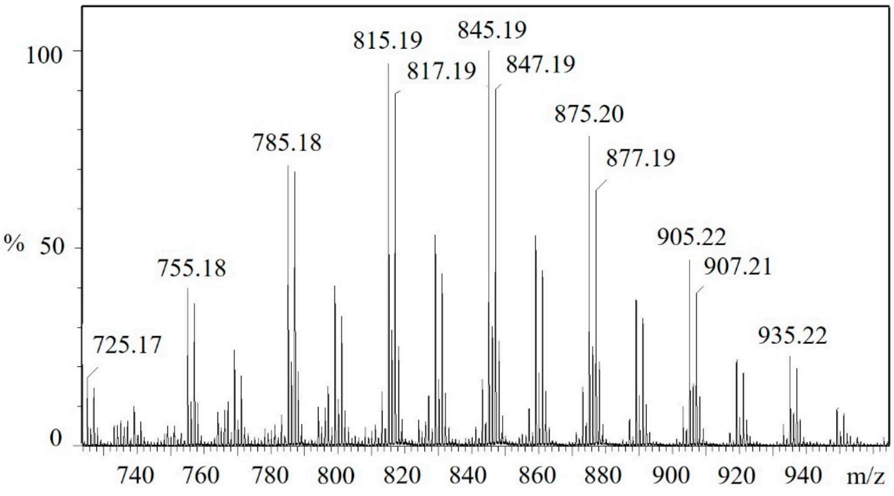

Figure S1. ESI mass spectrum of Ag^+ complex

Figure S1 shows the solution of Ag^+ in prepolymers characterized by electrospray ionization mass spectrometry (ESI-MS). It demonstrates a set of mass spectral peaks representing the formation of complexes between Ag^+ and melamine prepolymers, with all quasi-molecular ion peaks having the characteristics of Ag's A+2 isotope, which can be attributed to the complexation between different structural prepolymers monomers and Ag^+.

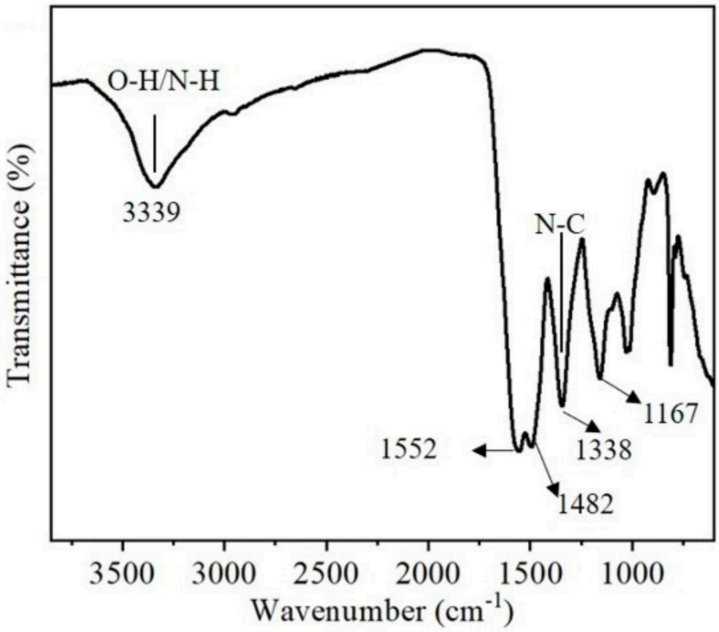

Figure S2. FT-IR spectra of Ag/MF colloidal nanospheres

Figure S2 presents the Fourier Transform Infrared (FT-IR) spectra of Ag/MF colloidal nanospheres. The absorption peak at 3339 cm-1 corresponds to the stretching vibration of O-H or N-H bonds, indicating the presence of -NH- groups formed through addition reactions between imine and amino groups within the microspheres. The two absorption peaks at 1552 cm-1 and 1489 cm-1 are characteristic of triazine ring vibrations. The absorption peak at 1338 cm-1 is associated with O-H and C-N bonds in the prepolymers. Due to the presence of sulfonic acid groups in 2,4-diaminobenzenesulfonic acid, an absorption peak at 1167 cm-1 corresponding to S=O vibrations is observed. This also suggests that 2,4-diaminobenzenesulfonic acid not only participates in the reaction but also catalyzes the condensation between the silver complex and the melamine prepolymer.

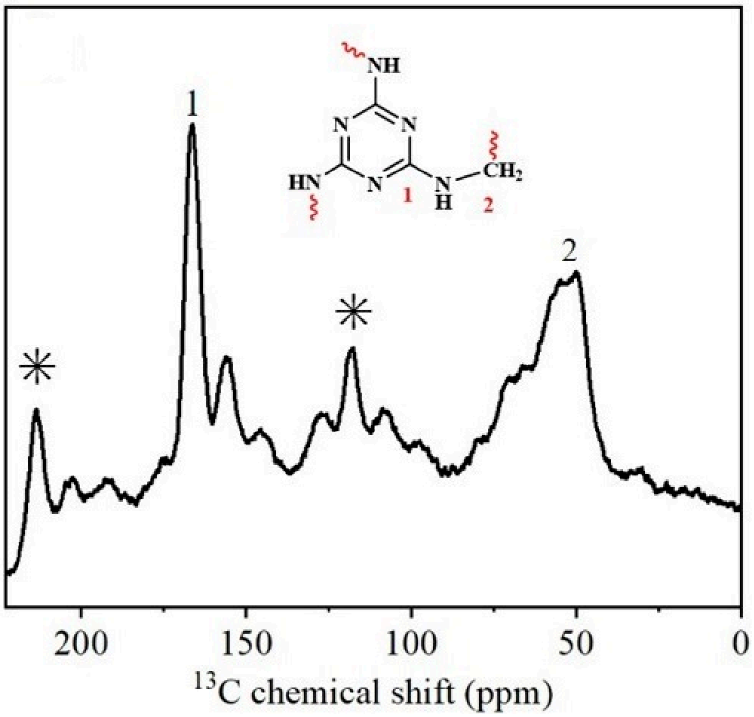

Figure S3. ^{13}C NMR spectra of Ag/MF colloidal nanospheres

Figure S3 presents the ^{13}C NMR spectrum of Ag/MF nanospheres, indicating the structural unit formed by 2,4-diaminobenzenesulfonic acid, silver, and melamine prepolymer. The peak at 168 ppm corresponds to the carbon atoms of melamine, while the peak at 54.8 ppm represents the conversion of the C(2) methylene carbon atom from the prepolymer's hydroxyethyl group through condensation reaction. These observations suggest that 2,4-diaminobenzenesulfonic acid catalyzes the polymerization of the prepolymer, confirming its role as a nanocatalyst in this system.

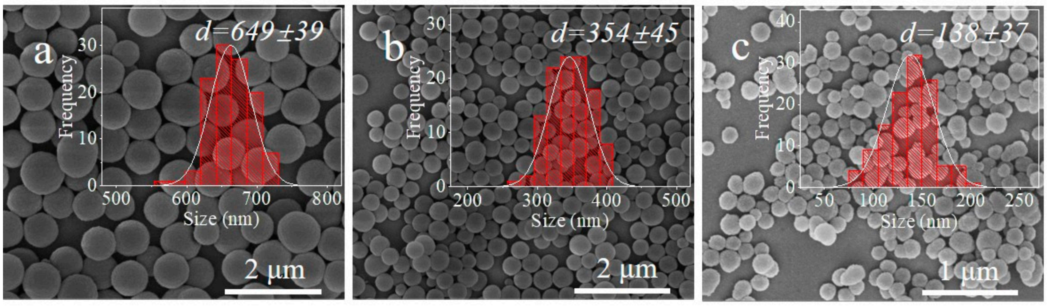

Figure S4. SEM images of Ag/MF colloidal nanospheres at different concentrations of 2,4-diamino-benzenesulfonic acid (a: Ag/MF-1, b: Ag/MF-2, c: Ag/MF-3)

Figure S4 presents the scanning electron microscopy (SEM) images of Ag/MF colloidal nanospheres characterized at varying amounts of 2,4-diaminobenzenesulfonic acid addition. The images reveal that with the increase in the concentration of 2,4-diaminobenzenesulfonic acid, the size of the colloidal nanospheres progressively diminishes, with Ag/MF-1, Ag/MF-2, and Ag/MF-3 displaying mean sizes of 649±39 nm (Figure S3a), 354±45 nm (Figure 3Sb), and 138±37 nm (Figure 3Sc), respectively. This size reduction is attributed to the influence of 2,4-diaminobenzenesulfonic acid on the reaction rate during the nucleation stage of the colloidal microspheres and the intermolecular repulsive forces, which in turn affect the number of nucleation events and ultimately determine the growth size of the colloidal microspheres. Despite altering the particle size of Ag/MF colloidal nanospheres, the addition of 2,4-diaminobenzenesulfonic acid does not modify their external morphology, maintaining a high uniformity and high dispersibility as spherical shapes.

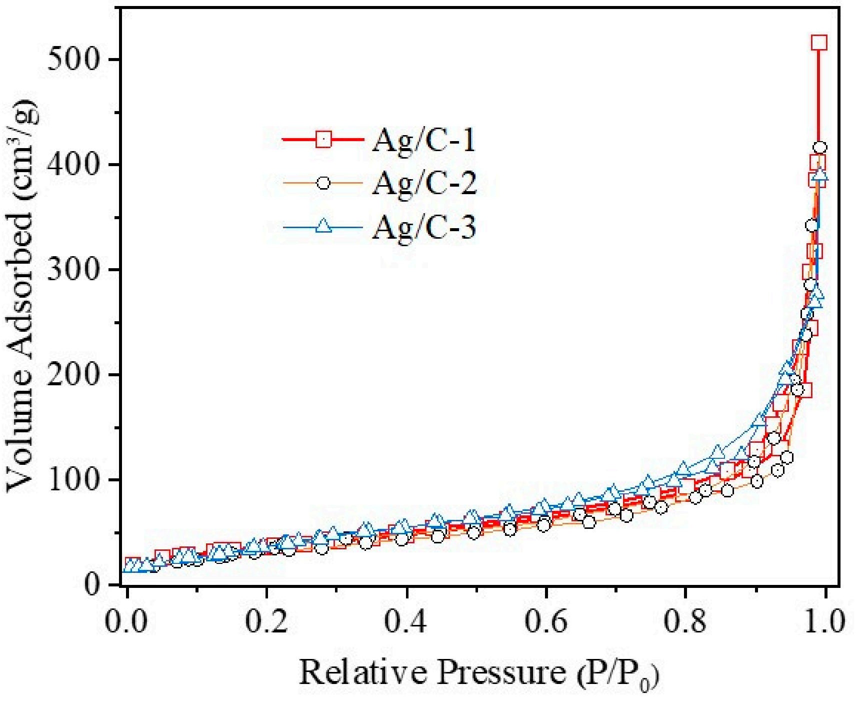

Figure S5. N_2 adsorption/desorption isotherms of Ag/C.

Figure S5 shows the N_2 adsorption/desorption isotherms of catalysts Ag/C, and all three catalysts exhibit type II isotherms, representing non porous adsorption materials. Due to the strong interaction between the adsorbate and the surface, the adsorption capacity rapidly increases at lower relative pressures, resulting in a convex curve. The inflection point of the isotherm usually occurs near single-layer adsorption, and as the relative pressure continues to increase, multi-layer adsorption gradually forms. When reaching saturation vapor pressure, there are infinite adsorption layers.

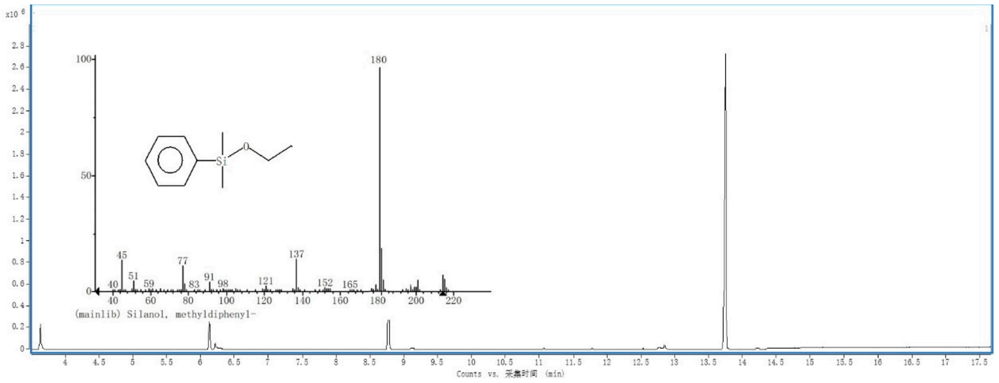

Figure S6. GS-MS of Si-O coupling reaction.

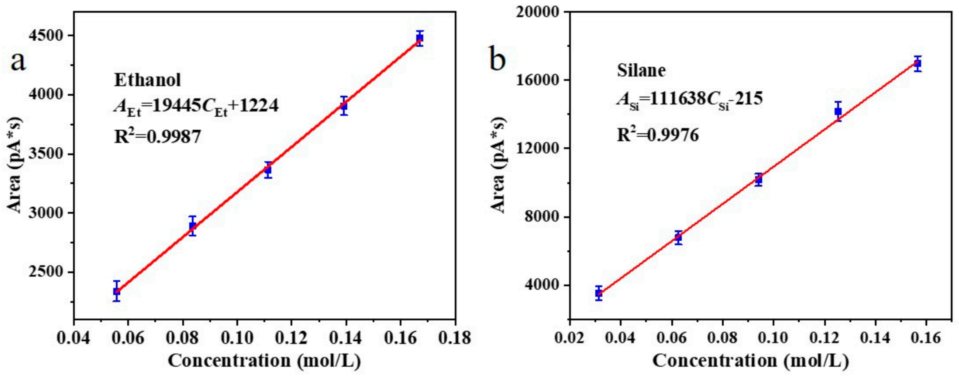

Figure S7. Kinetic plots of ethanol (a) and silane (b) in Si-O coupling reaction

Fig. S7a is a standard working curve of ethanol gas chromatography peak area to concentration, and the relationship is as follows:

$$A_{\mathrm{Et}}=19445C_{\mathrm{Et}}+1224 \qquad (1)$$

Where A_{Et} represents the ethanol vapor phase peak area, unit pA*s; C_{Et} denotes ethanol concentration in mol/L. The linear correlation coefficient was 0.9987, indicating that there was a good linear relationship between ethanol concentration and gas chromatographic peak area.

Fig. S7b shows the standard working curve of dimethylphenylsilane peak area to concentration, and the relationship is as follows:

$$A_{\mathrm{Si}}=111638C_{\mathrm{Si}}-215 \qquad (2)$$

A_{Si} denotes dimethylphenylsilane vapor peak area in pA*s; C_{Si} denotes dimethylphenylsilane concentration in mol/L with a linear correlation coefficient of 0.9976.

Supporting Tables (S1 to S5)

Table S1. Combustion elemental analysis of Ag/C.

No.	Name	Weight [mg]	Method	N [%]	C [%]	H [%]	S [%]	C/N ratio
1	Ag/C-1	2.804	5 mg 90s	29.43	57.12	3.135	0.567	1.94
2	Ag/C-2	3.203	5 mg 90s	27.51	58.09	2.627	0.193	2.11
3	Ag/C-3	2.755	5 mg 90s	28.92	58.35	2.237	0.432	2.01

Table S2. Specific surface area and porosity of Ag/C

Entry	Catalyst	Specific surface area	Pore volume	Average aperture

		(m^2g^{-1})	(m^3g^{-1})	(nm)
1	Ag/C-1	130.8	0.49	18.8
2	Ag/C-2	126.7	0.24	7.6
3	Ag/C-3	100.7	0.19	6.2

Table S3 Summary kinetic parameters for zero-grade Kinetic model

Reactant	Catalysts	*K* ± S.E.a	C ± S.E.a	R^2
Ethanol	Ag/C -1	-0.0008554±0.000033	0.1104±0.0024	0.9911

Ethanol	Ag/C -2	-0.0005789±0.000022	0.1106±0.0016	0.9917
Ethanol	Ag/C -3	-0.0004081±0.000019	0.1101±0.0014	0.9871
Silane	Ag/C -1	-0.0008796±0.000023	0.1104±0.0017	0.9958
Silane	Ag/C -2	-0.0004542±0.000016	0.1079±0.0011	0.9924
Silane	Ag/C -3	-0.0004015±0.000017	0.1129±0.0012	0.9884

a standard errors (S.E.)

Table S4. Adsorption amount of ethanol over Ag/C

Entry	Catalyst	Before adsorption (mol/L)	After adsorption (mol/L)	Adsorption capacity (mmol/g)
1	Ag/C-1	0.105	0.094	1.113
2	Ag/C-2	0.118	0.105	1.082

3	Ag/C-3	0.098	0.086	1.041

Table S5. Adsorption amount of silane over Ag/C

Entry	Catalyst	Before adsorption (mol/L)	After adsorption (mol/L)	Adsorption capacity (mmol/g)
1	Ag/C-1	0.126	0.110	1.554
2	Ag/C-2	0.125	0.109	1.491
3	Ag/C-3	0.122	0.109	1.413

Detection conditions: Tetrahydrofuran 6.0 mL, dimethylphenylsilane 0.1 mL, catalyst 20 mg, stirring at room temperature, gas chromatography to detect the adsorption amount, the internal standard is n-heptane.
